# Evaluating Support for the Current Classification of Eukaryotic Diversity

**DOI:** 10.1371/journal.pgen.0020220

**Published:** 2006-12-22

**Authors:** Laura Wegener Parfrey, Erika Barbero, Elyse Lasser, Micah Dunthorn, Debashish Bhattacharya, David J Patterson, Laura A Katz

**Affiliations:** 1 Program in Organismic and Evolutionary Biology, University of Massachusetts, Amherst, Massachusetts, United States of America; 2 Department of Biological Sciences, Smith College, Northampton, Massachusetts, United States of America; 3 Department of Biological Sciences, University of Iowa, Iowa City, Iowa, United States of America; 4 Roy J. Carver Center for Comparative Genomics, University of Iowa, Iowa City, Iowa, United States of America; 5 Bay Paul Center for Genomics, Marine Biological Laboratory, Woods Hole, Massachusetts, United States of America; University of Texas, United States of America

## Abstract

Perspectives on the classification of eukaryotic diversity have changed rapidly in recent years, as the four eukaryotic groups within the five-kingdom classification—plants, animals, fungi, and protists—have been transformed through numerous permutations into the current system of six “supergroups.” The intent of the supergroup classification system is to unite microbial and macroscopic eukaryotes based on phylogenetic inference. This supergroup approach is increasing in popularity in the literature and is appearing in introductory biology textbooks. We evaluate the stability and support for the current six-supergroup classification of eukaryotes based on molecular genealogies. We assess three aspects of each supergroup: (1) the stability of its taxonomy, (2) the support for monophyly (single evolutionary origin) in molecular analyses targeting a supergroup, and (3) the support for monophyly when a supergroup is included as an out-group in phylogenetic studies targeting other taxa. Our analysis demonstrates that supergroup taxonomies are unstable and that support for groups varies tremendously, indicating that the current classification scheme of eukaryotes is likely premature. We highlight several trends contributing to the instability and discuss the requirements for establishing robust clades within the eukaryotic tree of life.

## Synopsis

Evolutionary perspectives, including the classification of living organisms, provide the unifying scaffold on which biological knowledge is assembled. Researchers in many areas of biology use evolutionary classifications (taxonomy) in many ways, including as a means for interpreting the origin of evolutionary innovations, as a framework for comparative genetics/genomics, and as the basis for drawing broad conclusions about the diversity of living organisms. Thus, it is essential that taxonomy be robust. Here the authors evaluate the stability of and support for the current classification system of eukaryotic cells (cells with nuclei) in which eukaryotes are divided into six kingdom level categories, or supergroups. These six supergroups unite diverse microbial and macrobial eukaryotic lineages, including the well-known groups of plants, animals, and fungi. The authors assess the stability of supergroup classifications through time and reveal a rapidly changing taxonomic landscape that is difficult to navigate for the specialist and generalist alike. Additionally, the authors find variable support for each of the supergroups in published analyses based on DNA sequence variation. The support for supergroups differs according to the taxonomic area under study and the origin of the genes (e.g., nuclear, plastid) used in the analysis. Encouragingly, combining a conservative approach to taxonomy with increased sampling of microbial eukaryotes and the use of multiple types of data is likely to produce a robust scaffold for the eukaryotic tree of life.

## Introduction

Biological research is based on the shared history of living things. Taxonomy—the science of classifying organismal diversity—is the scaffold on which biological knowledge is assembled and integrated into a cohesive structure. A comprehensive eukaryotic taxonomy is a powerful research tool in evolutionary genetics, medicine, and many other fields. As the foundation of much subsequent research, the framework must, however, be robust. Here we test the existing framework by evaluating the support for and stability of the classification of eukaryotic diversity into six supergroups.

Eukaryotes (organisms containing nuclei) encompass incredible morphological diversity from picoplankton of only two microns in size to the blue whale and giant sequoia that are eight orders of magnitude larger. Many evolutionary innovations are found only in eukaryotes, some of which are present in all lineages (e.g., the cytoskeleton, nucleus) and others that are restricted to a few lineages (e.g., multicellularity, photosynthetic organelles [plastids]). These and other eukaryotic features evolved within microbial eukaryotes (protists) that thrived for hundreds of millions of years before they gave rise independently to multicellular eukaryotes, the familiar plants, animals, and fungi [1]. Thus, elucidating the origins of novel eukaryotic traits requires a comprehensive phylogeny—an inference of organismal relationships—that includes the diverse microbial lineages.

**Figure 1:**
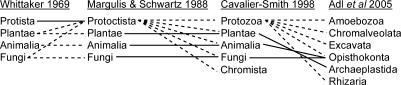
Trends in the Taxonomy of Eukaryotes A comparison of four representative taxonomies illustrates trends within eukaryotic taxonomy over the past 50 years [2,5–7]. Movement of taxa is traced from earlier to more recent taxonomies with solid and dashed lines. A solid line indicates all members of a group (left of line) are incorporated into the subsequent group (right of line). Dashed lines indicate that a subset of members (left) is incorporated into subsequent groups (right).

Higher-level classifications have historically emphasized the visible diversity of large eukaryotes, as reflected by the establishment of the plant, animal, and fungal kingdoms. In these schemes the diverse microbial eukaryotes have generally been placed in one (Protista [2–4] or Protoctista [5]) or two (Protozoa and Chromista [6]) groups (Figure 1; but see also [7,8]). However, this historic distinction between macroscopic and microscopic eukaryotes does not adequately capture their complex evolutionary relationships or the vast diversity within the microbial world.

In the past decade, the emphasis in high-level taxonomy has shifted away from the historic kingdoms and toward a new system of six supergroups that aims to portray evolutionary relationships between microbial and macrobial lineages. The supergroup concept is gaining popularity as evidenced by several reviews [9,10] and inclusion in forthcoming editions of introductory biology textbooks. In addition, the International Society of Protozoologists recently proposed a formal reclassification of eukaryotes into six supergroups, though acknowledging uncertainty in some groups [7].

### The Supergroups

**Figure 2:**
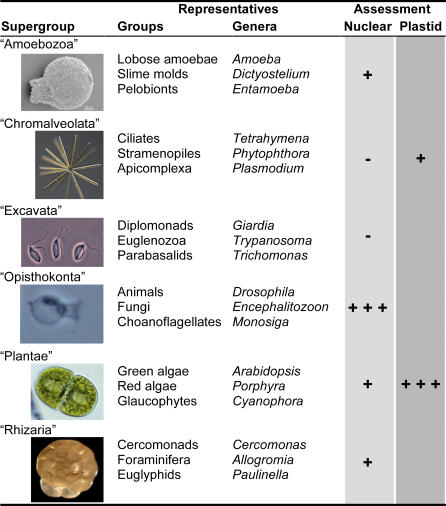
Summary of Eukaryotic Supergroups Assessment based on our analysis of molecular genealogies. +++, well supported; +, some support; −, support missing or very limited. Nuclear, genealogies based on nuclear genes. Plastid, genealogies based on chloroplast genes. Pictured organisms: *Lesquereusia, Thalassionema, Jakoba, Proterospongia, Cosmarium, Ammonia.* (Images: micro*scope, http://starcentral.mbl.edu/microscope).

Below we introduce the six supergroups in alphabetical order (Figure 2). The supergroup “Amoebozoa” was proposed in 1996 [11]. Original evidence for the group was drawn from molecular genealogies and morphological characters such as eruptive pseudopodia and branched tubular mitochondrial cristae. However, no clear synapomorphy—shared derived character—exists for “Amoebozoa.” In fact, amoeboid organisms are not restricted to the “Amoebozoa,” but are found in at least four of the six supergroups.

The “Amoebozoa” include a diversity of predominantly amoeboid members such as Dictyostelium discoideum (cellular slime mold), which is a model for understanding multicellularity [12]. Another member, *Entamoeba histolytica,* is an amitochondriate amoeba (Pelobiont) and is the cause of amoebic dysentery, an intestinal infection with global health consequences [13].

“Chromalveolata” was introduced as a parsimonious, albeit controversial, explanation for the presence of plastids of red algal origin in photosynthetic members of the “Alveolata” and “Chromista” [14]. Under this hypothesis, the last common ancestor of the chromalveolates was a heterotroph that acquired photosynthesis by engulfing a red alga and retaining it as a plastid [15,16]. The “Alveolata” include ciliates, dinoflagellates, and apicomplexa, and its monophyly is well supported by morphology and molecules. “Chromista” was created as a kingdom to unite diverse microbial lineages with red algal plastids (and their nonphotosynthetic descendants) [6,17], but no clear synapomorphy unites this clade.

The supergroup “Chromalveolata” includes microbes with critical roles in the environment and in human health. Numerous key discoveries emerged from studies of the model organism *Tetrahymena* (ciliate: “Alveolata”), including self-splicing RNAs and the presence of telomeres [18]. *Phytophthora* (stramenopile: “Chromista”), a soil-dwelling organism, is the causative agent of the Irish Potato Famine [19], whereas *Plasmodium* (Apicomplexa: “Alveolata”) is the causative agent of malaria [20].

“Excavata” is a supergroup composed predominately of heterotrophic flagellates whose ancestor is postulated to have had a synapomorphy of a conserved ventral feeding groove [21]. Most members of “Excavata” are free-living heterotrophs, but there are notable exceptions that are pathogens. For example, *Giardia* (Diplomonada) causes the intestinal infection giardiasis, and Trichomonas vaginalis (Parabasalia) is the causative agent of a sexually transmitted disease [22]. Kinetoplastids, such as *Trypanosoma* (Euglenozoa), have unique molecular features such as extensive RNA editing of mitochondrial genes that is templated by minicircle DNA [23].

“Opisthokonta” includes animals, fungi, and their microbial relatives. This supergroup emerged from molecular gene trees [24] and is united by the presence of a single posterior flagellum in many constituent lineages [25]. Molecular studies have expanded microbial membership of the group and revealed a potential molecular synapomorphy, an insertion in the Elongation Factor 1α gene in lineages containing this ortholog [26,27].

“Opisthokonts” include many biological model organisms *(Drosophila, Saccharomyces).* Vast amounts of research have been conducted on members of this supergroup and much textbook science is based on inferences from these lineages. Other notable opisthokonts include *Encephalitozoon* (Microsporidia: Fungi), a causative agent of diarrhea, which has one of the smallest known nuclear genomes at 2.9 MB [28]. Also included within the “Opisthokonta” are the choanoflagellates (e.g., *Monosiga*), which are the sister to animals [29].

The supergroup “Plantae” was erected as a kingdom in 1981 [30] to unite the three lineages with primary plastids: green algae (including land plants), rhodophytes, and glaucophytes. Under this hypothesis a single ancestral primary endosymbiosis of a cyanobacterium gave rise to the plastid in this supergroup [31]. The term “Plantae” has been used to describe numerous subsets of photosynthetic organisms, but in this manuscript will only be used in reference to the supergroup.

Well-known “Plantae” genera include *Arabidopsis,* a model angiosperm, and *Porphyra* (red alga), the edible seaweed nori. Within the “Plantae” there have been numerous independent origins of multicellularity including: *Volvox* (Chlorophyta) [32], the land plants, and red algae.

“Rhizaria” emerged from molecular data in 2002 to unite a heterogeneous group of flagellates and amoebae including: cercomonads, foraminifera, diverse testate amoebae, and former members of the polyphyletic radiolaria [33]. “Rhizaria” is an expansion of the “Cercozoa” [6] that was also recognized from molecular data [34,35]. “Cercozoa” and foraminifera appear to share a unique insertion in ubiquitin [36], but there is a paucity of non-molecular characters uniting members of “Rhizaria.”

“Rhizaria” encompasses a diversity of forms, including a heterotrophic flagellate *Cercomonas* (Cercomonada: “Cercozoa”) and a photosynthetic amoeba *Paulinella chromatophora,* (Silicofilosea: “Cercozoa”). The latter likely represents a recent endosymbiosis of a cyanobacterium [37,38]. Some members of the “Rhizaria,” notably the shelled foraminifera, also have a substantial fossil record that can be used to determine the age of sediments [39].

### Our Approach

**Figure 3:**
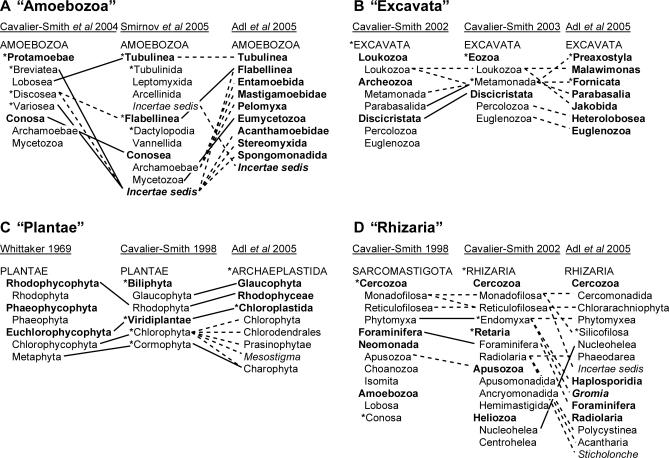
Trends in Supergroup Taxonomy A comparison of three formal classifications illustrates trends within (A) “Amoebozoa” [7,45,47]; (B) “Excavata” [7,33,60]; (C) “Plantae” [2,6,7]; and (D) “Rhizaria” [6,7,33]. A majority of solid, horizontal lines would indicate temporal stability of supergroup classification. For visual simplicity we do not indicate groups newly included in the supergroups or taxonomic restructuring within subgroups. Asterisk indicates a newly introduced term. “Chromalveolata” and “Opisthokonta” are not included because only one formal taxonomy exists for both groups. See Figure 1 for further notes.

To assess the robustness of the six proposed supergroups, we compare formal taxonomies and track group composition and nomenclature across time (Figures 1 and 3). We also evaluate support for the six supergroups by analyzing published molecular genealogies that either target a specific supergroup or aim to survey all supergroups. Our focus on molecular genealogies is limited. We recognize that supergroups have, in many cases, been defined by suites of characters such as flagellar apparatus in “Excavata” [33,40] and “Opisthokonta”[25], and that groups are more robust when supported by multiple data types (see Discussion). Use of genealogies is further complicated because a genealogy is the reconstruction of the history of a gene, and may or may not be congruent with phylogenies, which depict the history of organisms [41,42]. Despite these factors, our treatment of molecular genealogies is warranted given the prevalence of molecular analyses in the literature that seeks support for supergroups and the reliance on these gene trees in establishing taxonomy.

**Figure 4:**
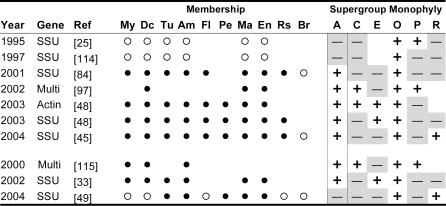
Support for Membership and Supergroup Monophyly from “Amoebozoa”-Targeted Molecular Genealogies Membership: • indicates the member taxon falls within the supergroup Amoebozoa; ○ indicates that the member taxon is excluded from the Amoebozoa clade, or no clade is formed. Papers below blank line survey eukaryotic diversity [33,49,115] and are included in all analyses. Member taxa: My, Mycetozoa; Dc, Dictyosteliids; Tu, Tubulinea (Lobosea, Gymnamoebea *sensu stricto*); Am, Acanthamoebidae; Fl, Flabellinea (Discosea, Glycostylea); Pe, *Pelomyxa*; Ma, Mastigamoebidae; En, Entamoebidae; Rs, residua; Br, *Breviata,* “*Mastigamoeba invertans sensu* NCBI.” Supergroup Monophyly, + indicates monophyly; − indicates group is para- or polyphyletic, and blank indicates insufficient data available. Supergroup definition based on Adl et al. 2005 [4]: A, Amoebozoa; C, Chromalveolata; E, Excavata; O, Opisthokonta; P, Plantae; R, Rhizaria. The position of *Breviata,* Br, was not considered when scoring the monophyly of Amoebozoa as this organism was misidentified and affiliations are unknown (see text). Some nodes were constrained in reference [97]. References cited in this figure are [25,33,45,48,49,84,97,114,115].

**Figure 5:**
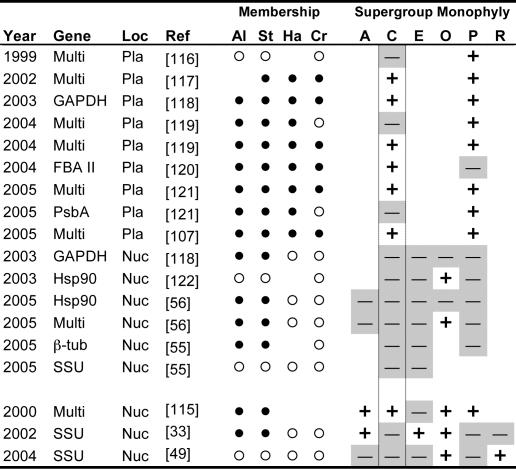
Support for Membership and Supergroup Monophyly from “Chromalveolata”-Targeted Molecular Genealogies Member taxa: Al, Alveolata; St, Stramenopiles (Heterokonts); Ha, Haptophyta; Cr, Cryptophyceae. Monophyletic “Plantae” from plastid genealogies includes secondarily derived plastids. See Figure 4 for further notes. References cited in this figure are [33,49,55,56,107,108,115–122]. Loc, location (genome) from which the gene of interest originated; Pla, plastid genome; Nuc, nuclear genome; Mit, mitochondrial genome.

**Figure 6:**
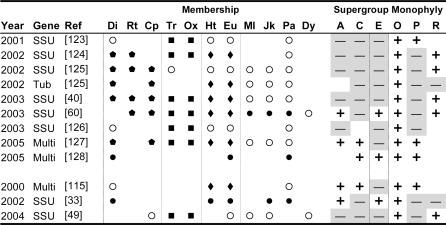
Support for Membership and Supergroup Monophyly from “Excavata”-Targeted Molecular Genealogies Member taxa: Di, Diplomonadida; Rt, Retortamonadida; Cp, *Carpediemonas*; Tr, *Trimastix*; Ox, Oxymonadida; Ht, Heterolobosea; Eu, Euglenozoa; Ml, *Malawimonas;* Jk, Jakobida; Pa, Parabasalia; Dy, *Diphylleia.* Hypothesized subgroups: 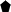 Fornicata clade (Di + Rt + Cp) monophyletic, Preaxostyla clade (Ox + Tr) monophyletic, ♦ Discicristata clade (Ht + Eu) monophyletic. The position of *Diphylleia,* Dy, was not considered when scoring the monophyly of “Excavata” as the inclusion of this organism within “Excavata” is controversial and has been removed from recent classifications (see text). See Figure 4 for further notes. References cited in this figure are [33,40,49,60,115,123–128].

**Figure 7 pgen-0020220-g007:**
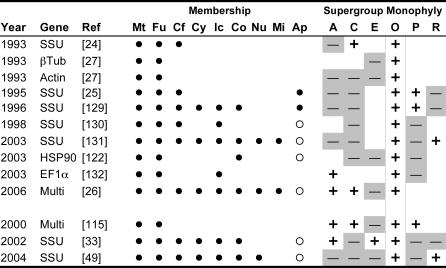
Support for Membership and Supergroup Monophyly from “Opisthokonta”-Targeted Molecular Genealogies Member taxa: Mt, Metazoa; Fu, Fungi; Cf, Choanomonada; Cy, chytrids; Ic, Ichthyosporea (DRIPs); Cl, *Corallochytrium;* Nu, Nucleariida; Mi, *Ministeria*; Ap, apusomonads. The position of apusomonads*,* Ap, was not considered when scoring the monophyly of “Opisthokonta” as this organism is highly variable, and it has been removed from recent classifications (see text). See Figure 4 for further notes. References cited in this figure are [24–27,33,49,115,122,129–132].

**Figure 8:**
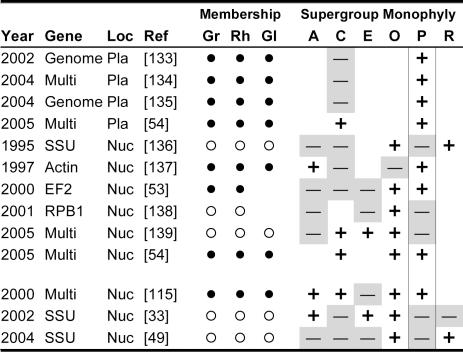
Support for Membership and Supergroup Monophyly from “Plantae”-Targeted Molecular Genealogies Member taxa: Gr, Chloroplastida = Viridiplantae (Green algae, including land plants); Rd, Rhodophyceae (Red algae); Gl, Glaucophyta. See Figure 4 for general notes and Figure 5 for plastid-specific notes. References cited in this figure are [33,49,53,54,115,133–139].

**Figure 9:**
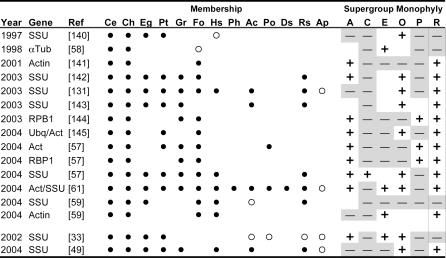
Support for Membership and Supergroup Monophyly from “Rhizaria”-Targeted Molecular Genealogies Member taxa: Ce, Cercomonadida; Ch, Chlorarachniophyta; Eg, euglyphids; Pt, Phytomyxea (plasmophorids); Ph, Phaeodarea; Gr, *Gromia;* Fo, Foraminifera; Hs, Haplosporidia (Ascetosporea); Po, Polycystinea; Ac, Acantharia; Ds, desmothoracids; Rs, residua; Ap, apusomonads. The position of apusomonads*,* Ap, was not considered when scoring the monophyly of “Rhizaria” as the position of this organism is highly variable, and it has been removed from recent classifications (see text). See Figure 4 for further notes. References cited in this figure are [33,49,57–59,61,131,140–145].

For each genealogy we evaluate the taxon sampling for the targeted supergroup (Membership; Figures 4–9) and the monophyly of all supergroups with at least two member taxa (Supergroup monophyly; Figures 4–9). Monophyletic clades, those that include an ancestor and all of its descendants [43], are scored (+; Figures 4–9). We assess support for supergroups when they are targeted by specific studies and when they are included as out-groups in studies targeting other supergroups. A conservative measure of out-group monophyly was used because we required only two member lineages be present. In contrast, focal supergroups had broader taxonomic sampling.

## Results

### Taxonomic Instability

There is considerable instability in taxonomies of the six putative supergroups (Figure 3). Causes of the rapid revisions in eukaryotic taxonomy over short time periods include: (1) nomenclatural ambiguity, (2) ephemeral and poorly supported higher-level taxa, and (3) classification schemes erected under differing taxonomic philosophies. For example, taxonomy of the “Amoebozoa,” a term originally introduced by Lühe in 1913 [44] to encompass a very different assemblage of organisms, has changed considerably in ten years (Figure 3A). “Variosea” was created as a subclade within the “Amoebozoa” in 2004 to group taxonomically unplaced genera of amoebae with “exceptionally varied phenotype” [45]. Rarely supported by morphology or molecular evidence [46–49], this taxon was excluded from subsequent classifications [7,47] but is still discussed in the literature [46]. Similarly, the excavate taxon “Loukozoa” [6] has been continually redefined to include a variety of taxa bearing a ventral groove (Figure 3B) and finally abandoned [40]. The taxonomy of “Rhizaria” has emerged largely from molecular genealogies and has varied partly in response to shifting topology of gene trees that change with taxon sampling and the method of tree construction [6,33,50,51] (Figure 3D).

The taxonomy of “Plantae” is destabilized by the complex history of the term. Used since Haeckel's time [52], “Plantae” has been redefined numerous times to describe various collections of photosynthetic organisms, leading to major discrepancies between taxonomic schemes (Figure 3C; e.g., [2,5]). The term “Archaeplastida” was recently introduced to alleviate confusion over “Plantae,” but this synonym is not widely used.

The stability of two supergroups, “Chromalveolata” and “Opisthokonta,” cannot be assessed at this time because only a single formal taxonomy exists [7]. Other classification schemes of eukaryotes segregate animals and fungi as separate kingdoms and place microbial opisthokonts in the kingdom Protozoa (Figure 1) [6,33]. Similarly, chromalveolate members are often divided between the polyphyletic kingdoms “Chromista” and “Protozoa” (Figure 1) [33,49].

### Varying Support for Membership within and Monophyly of Targeted Supergroups

Several supergroups are generally well supported when targeted in molecular systematic studies. Strikingly, the monophyly of both the original and expanded “Opisthokonta” members is strongly supported in all investigations targeting the group (ten of ten, Figure 7). Two other supergroups are also well supported: “Rhizaria” monophyly is recovered in 11 of 14 studies focusing on this supergroup (Figure 9) and “Amoebozoa” retained in five of seven topologies (Figure 4). However, support for these groups is expected, given that they were recognized from molecular gene trees [11,33].

“Excavata” rarely form a monophyletic group in molecular systematic studies targeting this supergroup (two of nine; Figure 6). Moreover, the position of putative members, jakobids, *Malawimonas,* parabasalids, and *Diphylleia* vary by analysis (Figure 6). Three distinct subclades, all of which are supported by ultrastructural characters [40], are generally recovered (Fornicata [six of six], Preaxostyla [six of six], and Discicristata [five of eight]; Figure 6).

Support for two supergroups varies depending on the type of character used: plastid or nuclear. The monophyly of “Plantae” and “Chromalveolata” are well supported by plastid characters: four of four plastid analyses (Figure 8) and six of nine (Figure 5), respectively. The “Plantae” clade is monophyletic in only three of six analyses using nuclear genes, including Elongation Factor 2 [53] and a 100+ gene analysis that included very limited taxon sample [54]. Nuclear loci never support “Chromalveolata” (zero of six; Figure 5), though alveolates and stramenopiles often form a clade to the exclusion of haptophytes and cryptophytes (e.g., [24,97]; Figures 4 and 7[Fig pgen-0020220-g007]).

### Decreased Support for Monophyly of Supergroups as Out-Groups in Other Studies

For each genealogy we also assessed the monophyly of the supergroups when included as out-groups. Overall, we find that support for the monophyly of a given supergroup is stronger when targeted and support decreases when the same supergroup is included as an out-group in other studies.

This trend is particularly unexpected given our less stringent requirements for monophyly of out-groups: a minimum of only two members need be included, while targeted groups had broader taxon sampling (see Methods). A priori, it would seem that the lower stringency could allow a limited sample of supergroup members to substitute for overall supergroup monophyly, thereby increasing the occurrence of supergroup monophyly for out-group taxa. However, this scenario is realized only in the groups that receive poor support, “Excavata” and “Chromalveolata,” assessed by nuclear genes. “Excavata” is monophyletic more frequently when members are included as out-groups (seven of 30, Figures 4, 5, and 7–9, versus two of nine, Figure 8). Taxonomic sampling of these lineages is often considerably lower in non-targeted analysis, and monophyly reflects that of the subclades “Discicristata” or “Fornicata” (such as in [48,58,59], but see [60,128] for two exceptions, Figures 4, 6, and 9). “Chromalveolata” is monophyletic in ten of 45 nuclear gene trees targeting other taxonomic areas (Figures 4 and 6–9). Intriguingly, in all ten of the cases where nuclear genes support monophyletic “Chromalveolata,” only alveolates and stramenopiles are included (Figures 4–9).

In contrast, the remaining supergroups are monophyletic less often when included as out-groups. For example, “Opisthokonta” was recovered in all studies targeting this supergroup, but in only 33 of 41 studies that target other groups (Figures 5–9). Similarly, both the “Amoebozoa” and “Rhizaria” are monophyletic less often when their members are included as out-groups in studies targeting the remaining five supergroups (15 of 35 and eight of 15, respectively: Figures 5–9 and 4–8). When included as an out-group, “Plantae” plastids usually form a monophyletic clade (eight of nine analyses, Figure 5) but support is much lower in nuclear gene trees (11 of 42, Figures 4–7 and 9).

## Discussion

Our analysis reveals varying levels of stability and support for the six supergroups (Figure 2). Below, we assess the status of each supergroup, describe factors that contribute to the instability, and propose measures to improve reconstruction of an accurate eukaryotic phylogeny.

### Supergroup Robustness

Robust taxa—those consistently supported by multiple datasets—are emerging and include the supergroup “Opisthokonta.” This group of animals, fungi, and their microbial relatives receives consistent support in molecular genealogies. This supergroup was monophyletic in 43 of 51 trees we examined (Figures 4–9). “Opisthokonta” is also united by additional types of data: most members share a single posterior flagellum, contain plate-like cristae in mitochondria, and have an insertion within the Elongation Factor 1α gene [8,25–27].

The remaining five supergroups receive varying degrees of support from molecular genealogies. “Amoebozoa” and “Rhizaria” received high support in analyses that targeted them (Figures 4 and 9, respectively) but formed monophyletic clades less often when included as out-groups. The two photosynthetic clades “Chromalveolata” and “Plantae” receive differential support depending on the origin of the gene: high support in plastid genealogies but low in nuclear gene trees (Figures 5 and 8, see Results). Molecular support for the “Excavata” as a whole is lacking from well-sampled gene trees (Figure 6).

Although the six supergroups are not consistently supported by molecular genealogies, some nested clades are emerging as robust groups. For example, a sister relationship between Alveolata and Stramenopila is often recovered. It is this relationship that makes “Chromalveolata” appear monophyletic in nuclear genealogies when only these clades are included as outgroups (e.g., [24,97], and Figures 4 and 7). There is also growing support for several subgroups within the poorly supported “Excavata” (i.e., “Fornicata” and “Preaxostyla”; Figure 6).

### Alternative Hypotheses

Although it is clear from our analysis that eukaryotic supergroups are not well supported, no alternative high-level groupings emerge from molecular genealogies. Rather, there is support for lower-level groups, such as the “Excavata” subgroups discussed above and perhaps also alveolates plus stramenopiles. This suggests that either there are no higher-level groupings to be found, or there is as yet inadequate data to resolve these clades. We believe that lack of taxon sampling is the key to resolution.

Further evidence against the six-supergroup view of eukaryotic diversity is the existence of “nomadic” taxa—lineages that do not have a consistent sister group, but instead wander between various weakly supported positions. Some nomadic taxa are acknowledged *incertae sedis* (of unknown taxonomic position) such as *Ancyromonas, Breviata,* and Apusomonadidae [7,8]. Other taxa that have been assigned to supergroups also appear to be nomadic, including Haptophyta (putative member of “Chromalveolata”) and *Malawimonas* (putative member of “Excavata”). For example, the haptophytes variously branch with Centrohelida and red algae [45], sister to a clade of “Rhizaria” and Heterolobosea [48], sister to cryptophytes [56], and in a basal polytomy [61]. These nomadic taxa may either represent independent, early diverging lineages or their phylogenetic position cannot yet be resolved with the data available. Again, we feel that taxon sampling is the key in order to distinguish between these possibilities.

### Why Is Eukaryotic Taxonomy So Difficult?

The variable support for relationships is in part attributable to the inherent difficulty of deep phylogeny, the chimeric nature of eukaryotes, misidentified organisms, and conflicting approaches to taxonomy. Here we elaborate on these destabilizing trends and provide illustrative examples.

#### Challenges of deep phylogeny.

Reconstructing the history of eukaryotic lineages requires extraction of phylogenetic signal from the noise that has accumulated over many hundreds of millions of years of divergent evolutionary histories. There is doubt whether resolution of divergences this deep can be resolved with molecular data [62]. Additionally, the nature of the relationships may also pose a significant challenge. For example, a rapid radiation of major eukaryotic lineages has been proposed [63] and is the most difficult scenario to resolve because of the lack of time to accumulate synapomorphies at deep nodes.

Further, phylogenetic relationships can be obscured by heterogeneous rates of evolution and divergent selection pressures. For example, genes in many parasitic lineages of eukaryotes experience elevated rates of evolution. If not properly accounted for, these fast lineages will group together due to long-branch attraction [64,65]. This was the case for Microsporidia, intracellular parasites of animals; early small subunit rDNA (SSU) genealogies placed the Microsporidia at the base of the tree with other amitochondriate taxa, including *Giardia* and *Entamoeba* [66]. These parasites were united under the “Archezoa” hypothesis [67]. More recent analyses with appropriate models of evolution [68] and those using protein-coding genes [69] place the Microsporidia within fungi and falsify “Archezoa.” This example demonstrates the importance of phylogenetic methods in the interpretation of eukaryotic diversity. In our analysis we find no clear correlation between method of tree building and group stability. Arguments about phylogenetic inference have been discussed extensively [62,70–76], and increasingly sophisticated algorithms are being developed to compensate for the difficulties [77–79].

#### The chimeric nature of eukaryotes.

Reconstructing the history of eukaryotic lineages is complicated by the horizontal transfer of genes and organelles [74,80–83]. For example, “Chromalveolata” plastid genes tell one story, consistent with a single transfer from red algae, which is not currently supported by available nuclear genes (Figure 5). There is also a growing body of evidence for aberrant lateral gene transfers in eukaryotes (reviewed in [80,82]).

#### Instability due to misidentification.

Misidentification destabilizes taxonomy because all efforts to classify a misidentified organism reach erroneous conclusions. Cases of misidentification lead to inaccurate conclusions and require considerable effort to remedy. There is a rigorous standard for identifying microbial eukaryotes, but this standard is not always upheld. For example, the putative “Amoebozoa” species *“Mastigamoeba invertens”* that always branched outside the “Amoebozoa” clade [45,49,84] was misidentified [85]; it has now been properly described as Breviata anathema and is not yet placed within any of the supergroups [85].

Inaccurate conclusions about organismal relationships can also result from contamination (e.g., from symbionts and parasites). The results of subsequent molecular genealogies are therefore wrong and misleading. For example, opalinids, multinucleated flagellates that inhabit the lower digestive track of Anurans, were placed in the stramenopiles (Slopalinida: “Chromalevolata”) based on ultrastructural data [86]. However, the first molecular sequences for this group placed them within fungi (Opalina ranarum and Cepedea virguloidea [87,88]). These sequences were later shown to belong to zygomycete fungal contaminants, not to the opalinids. Subsequent isolates *(Protoopalina intestinalis)* yielded genealogies congruent with the ultrastructural data, placing P. intestinalis within the stramenopiles [89]. To avoid setbacks and confusion due to misidentification, we propose that all analyses of eukaryotic diversity include a vouchering system for strains, images, and DNAs.

#### Conflicting approaches to taxonomy.

Our evaluation of the stability of taxonomy for supergroups reveals a rapidly changing landscape (Figures 1 and 3). The instability in higher-level classifications of eukaryotes reflects the diversity of philosophical approaches, the exploratory state of eukaryotic taxonomy, and premature taxon naming. Many researchers seek schemes based on monophyletic groupings so that their taxonomies reflect evolutionary relationships [7,8,90,91]. In contrast, others employ a taxonomic philosophy in which evolutionary relatedness and monophyly are just one criterion from a set of group characteristics [33]. Paraphyly—a taxon defined without all descendants—is tolerated in these systems, and paraphyletic taxa are designated as such (see [6] p. 210–215 for explanation of such a philosophy).

In many cases, classification schemes that are separated by two years or less vary substantially from one another (e.g., Figure 3A and 3B). New groups and fluctuating group composition result in numerous cases of homonymy (two concepts linked to one name), synonymy (one concept linked to two names), and redefinition of existing terms. For example, at the highest level the terms “Amoebozoa,” “Opisthokonta,” and “Plantae” were all introduced under different definitions [4,44,52] before being applied to supergroups. The term “Plantae” is an extreme case of homonymy having referred to numerous groups of photosynthetic organisms over the past century and a half (Figure 3C). The rapidly changing taxonomic landscape makes it difficult for non-specialists as well specialists to follow the current debate over supergroups.

### Toward a Robust Scaffold to the Eukaryotic Tree of Life

#### Taxonomic sampling.

Perhaps the most critical aspect of the current state of eukaryotic systematics is the very limited taxonomic sampling to date. This is particularly problematic as the supergroup literature is often derived from a resampled pool of genes and taxa. More than 60 lineages of microbial eukaryotes have been identified by ultrastructure [8], yet only about one-half of these have been included in molecular analyses. Furthermore, even when these lineages are included, they are generally represented by a single species. Such sparse sampling increases the risk of long-branch attraction as discussed above, such as occurred for *Giardia,* and may cause artifactual relationships [92]. Further, analyses of sequences from newly sampled lineages have altered or expanded supergroup definitions (e.g., nucleariids in “Opisthokonta” [93] and Phaeodarea in “Rhizaria” [94]). Thus, statements of monophyly may be premature when taxonomic sampling is low.

There is tension between increasing the number of taxa versus the numbers of genes. Several theoretical works have demonstrated the diminishing returns of increased number of genes relative to increased taxon sampling [95–97], but see [98]. In addition, increasing taxon sampling can lead to shifts in molecular tree topology [99–101]. These results provide incentive to concentrate sequencing efforts on obtaining more taxa and a moderate number of genes. We recommend increasing the lineages sampled and the number of diverse taxa within lineages. We are optimistic that as data become available from a greater diversity of taxa, eukaryotic phylogeny will become increasingly more resolved.

#### Multiple character sets.

We further anticipate that support for clades will increase as additional character sets are incorporated. Phylogenies based on single characters, whether genes, morphology, or ultrastructure, are subject to biases in the data and are not reliable by themselves. Hence, multiple character sets should be used to corroborate results. Ultrastructural apomorphies combined with molecular genealogies have proven to be good indicators of phylogeny at the level below supergroups [40,102]. This approach has bolstered support for “Fornicata” and “Preaxostyla,” which are consistently recovered in molecular genealogies and have defining ultrastructural characters. As we move forward with multiple character sets, we must shift from searching for characters to support hypotheses to evaluating hypotheses in light of all available data.

Well-sampled multigene and genome scale molecular systematics provide another powerful tool for resolving ancient splits in the tree of life. The National Science Foundation initiative “Assembling the Tree of Life” provides evidence of this shift in systematics research, whereby all proposals involve multigene or genome (organellar) sequencing to establish robust phylogenetic hypotheses (see http://www.nsf.gov/pubs/2005/nsf05523/nsf05523.htm; [54,97]). The EuTree consortium (http://www.eutree.org) aims to increase substantially the sampled diversity of eukaryotes by focusing on understudied lineages in our multigene project to assemble the tree of life.

An example of multigene study is analysis of genes involved in clade-specific functions. This approach has been employed in testing “Plantae” and “Chromalveolata” (e.g., [103]). A single endosymbiosis (of a cyanobacterium in “Plantae” and red alga in “Chromalveolata”) predicts that the systems that facilitate controlled exchange of metabolic intermediates between the symbiotic partners be shared by putative members of these two supergroups [104]. This prediction has been supported by analyses of the plastid import machinery [105] and antiporters that transport fixed carbons across the plastid membranes [106]. However, taxon sampling has been limited in these studies. Currently, increased sampling of genomes from diverse photosynthetic eukaryotes is yielding additional genes for clade-specific predictions [107,108].

#### A conservative approach to taxonomy.

Because taxonomy is the foundation for much of the dialog and research in evolutionary biology, there must be an unambiguous taxonomic system in which one term is linked to one concept. In contrast to this ideal, homonymy and redefinition are prevalent in the taxonomy of eukaryotes, often as the result of premature introduction or redefinition of taxa (see above; Figure 3). Emerging hypotheses benefit the community by sparking new research to test the hypothesis, but they also introduce ambiguity. To alleviate the confusion, we suggest introducing hypotheses as informal groups and using inverted commas to indicate the existence of a caveat, as done with the uncertain groups in this manuscript. These steps will inform the community that group composition is likely to change, alleviate quick taxon turnover, and promote stable taxa that are more resistant to compositional change.

As increasing amounts of data become available, well-supported nodes emerge and classifications tend to stabilize, such as is occurring for the ordinal framework for angiosperms [109,110]. Similarly, we expect that this conservative approach, combined with increased sampling of taxa and genes, will promote the future stabilization of eukaryotic classification.

### Conclusion

Although the level of support varies among groups, the current classification of eukaryotes into six supergroups is being adopted broadly by the biological community (i.e., evidenced by its appearance in biology textbooks). The supergroup “Opisthokonta” and a number of nested clades within supergroups are supported by most studies. However, support for “Amoebozoa,” “Chromalveolata,” “Excavata,” “Plantae,” and “Rhizaria” is less consistent. The supergroups, and eukaryotic taxonomy in general, are further destabilized by considerable fluidity of taxa, taxon membership, and ambiguous nomenclature as revealed by comparison of classification schemes.

The accurate reconstruction of the eukaryotic tree of life requires: (1) a more inclusive sample of microbial eukaryotes; (2) distinguishing emerging hypotheses from taxa corroborated by multiple datasets; and (3) a conservative, mutually agreed upon approach to establishing taxonomies. Analyses of these types of data from a broad, inclusive sampling of eukaryotes are likely to lead to a robust scaffold for the eukaryotic tree of life.

## Methods

### 

#### Stability of taxonomy.

To assess the stability of supergroup taxonomies over time, we selected three classification schemes for each supergroup and tracked both the stability of taxa membership (solid and dashed lines; Figures 1 and 3) and the fate of newly created taxon names (asterisk; Figure 3). In sampling representative taxonomies, we aimed to capture a diversity of authors and opinions. In the case of “Opisthokonta” and “Chromalveolata” we are aware of only one formal, peer-reviewed classification scheme [7]. Given the lack of equivalency in ranks between taxonomies, we have chosen to display three levels with the intention of listing equivalent levels clearly.

#### Membership support.

Within each supergroup, we assess the support for each member taxon by documenting its inclusion in molecular genealogies (Figures 4–9). Member taxa were chosen because they are historically a well-supported group, usually with an ultrastructural identity. The haptophytes are such a group, and share a haptonema [8]. We included members that represent a broad interpretation of the supergroup. For example, “Rhizaria” member taxa include groups (e.g., apusomonads) originally placed in “Rhizaria” but later removed. We considered a taxon to be a supported member of its supergroup (filled circles; Figures 4–9) when it falls within a monophyletic clade containing a majority of the supergroup members. A taxon that falls outside of its supergroup clade, or on the occasion that a majority of members do not form a monophyletic clade, is considered unsupported in that genealogy (open circles; Figures 4–9).

The inclusion of a genealogy requires that it be found in a paper that specifically addresses one of the supergroups or analyzes broad eukaryotic diversity. The genealogies must also include adequate sampling—two-member taxa per supergroup—from at least two of the six supergroups to allow for the comparison of supergroup monophyly. In cases where multiple gene trees are presented we display the authors' findings as multiple entries when the trees are not congruent or as a single entry when the trees are concordant. Due to the lack of monophyly in virtually all analyses, we have evaluated the support for several hypothesized subgroups within the “Excavata” (geometric shapes; Figure 6).

#### Supergroup monophyly.

To assess monophyly of supergroups, we used the set of genealogies described above to evaluate the molecular support for the supergroups as interpreted by Adl et al. 2005 ([7]; Figures 4–9). We analyzed the monophyly [43] of each supergroup in trees having at least two member taxa present (+/− Figures 4–9). We do not indicate the method of tree construction. Although the algorithm used is important, we did not find a clear correlation between supported groups and algorithm used. We were also liberal in accepting any level of support (e.g., bootstrap values and posterior probabilities ranged from 4%–100%) when determining monophyly, in part because there is debate over acceptable cutoff values [111–113].

## Supporting Information

### Accession Numbers

Information about commonly used genes for phylogenesis of microbial eukaryotes discussed in this paper can be found in the Homologene database at NCBI (http://www.ncbi.nlm.nih.gov/Genbank): actin (88645), α-tubulin (81745), β-tubulin (69099), Elongation Factor 1α gene (68181), small subunit rDNA (6629), and ubiquitin (39626). Accession numbers for genes from misidentified organisms can be found at NCBI in GenBank (http://www.ncbi.nlm.nih.gov/Genbank). Misidentified opalinids: Opalina ranarum (AF141969) and Cepedea virguloidea (AF141970); correctly identified Protoopalina intestinalis (AY576544–AY576546) and Breviata anathema (AF153206). Sequences for Encephalitozoon cuniculi can be found at NCBI under genome project number 9545.
